# Influence of type 2 diabetes mellitus on liver histology among morbidly obese individuals. A cross-sectional study: Influência do diabetes mellitus tipo 2 sobre a histologia hepática entre indivíduos com obesidade mórbida. Um estudo transversal

**DOI:** 10.1590/1516-3180.2015.01652409

**Published:** 2016-01-19

**Authors:** Everton Cazzo, Laísa Simakawa Jimenez, Fábio de Felice Gallo, José Carlos Pareja, Elinton Adami Chaim

**Affiliations:** I MD, MSc. Assistant Lecturer, Department of Surgery, Universidade Estadual de Campinas (Unicamp), Campinas, São Paulo, Brazil.; II BM. Medical Student, Department of Surgery, Universidade Estadual de Campinas (Unicamp), Campinas, São Paulo, Brazil.; III MD. Resident Physician, Department of Surgery, Universidade Estadual de Campinas (Unicamp), Campinas, São Paulo, Brazil.; IV MD, PhD. Adjunct Professor, Department of Surgery, Universidade Estadual de Campinas (Unicamp), Campinas, São Paulo, Brazil.; V MD, MSc, PhD. Associate Professor, Department of Surgery, Universidade Estadual de Campinas (Unicamp), Campinas, São Paulo, Brazil.

**Keywords:** Fatty liver, Insulin resistance, Diabetes mellitus, Bariatric surgery, Obesity

## Abstract

**CONTEXT AND OBJECTIVE:**

Nonalcoholic fatty liver disease (NAFLD) has become a public health concern. It encompasses a wide spectrum of histological abnormalities and has close relationships with insulin resistance and type 2 diabetes mellitus (T2DM). This study sought to compare the histological alterations observed in morbidly obese individuals with and without T2DM who underwent Roux-en-Y gastric bypass.

**DESIGN AND SETTING:**

Cross-sectional study in a tertiary-level public hospital.

**METHODS:**

This was a cross-sectional study on 197 individuals who underwent gastric bypass surgery between 2011 and 2013. NAFLD was assessed through liver biopsies. T2DM was diagnosed through the International Diabetes Federation criteria.

**RESULTS:**

Non-diabetics presented significantly more biopsies without any histological abnormalities, regarding steatosis (42.6% versus 25.5%; P = 0.0400), fibrosis (60.6% versus 36.2%; P = 0.0042) and steatohepatitis (27.3% versus 12.8%; P = 0.0495), while diabetics presented significantly higher frequency of moderate forms of steatosis (36.2% versus 20%; P = 0.0307) and fibrosis (23.4% versus 4%; P = 0.0002).

**DISCUSSION:**

T2DM was associated with more advanced forms of NAFLD within the population studied. NAFLD has previously been correlated with severe forms of heart disease.

**CONCLUSION:**

Screening for and early detecting of NAFLD in high-risk populations are important for avoiding further development of severe forms and the need for liver transplantation.

## RESUMO

**CONTEXTO E OBJETIVO:**

A doença gordurosa não alcoólica do fígado se tornou um problema de saúde pública. Ela compreende um amplo espectro de alterações histológicas e se relaciona intimamente com a resistência insulínica e o diabetes mellitus tipo 2. Este estudo tem por objetivo comparar as alterações histológicas observadas em obesos mórbidos com e sem diabetes submetidos ao *bypass* gástrico em Y de Roux.

**TIPO DE ESTUDO E LOCAL:**

Estudo transversal em um hospital terciário público.

**MÉTODOS:**

Este é um estudo transversal que envolveu 197 indivíduos submetidos ao *bypass* gástrico entre 2011 e 2013. A doença gordurosa do fígado foi avaliada através de biópsia hepática. O diabetes mellitus tipo 2 foi diagnosticado através dos critérios da Federação Internacional de Diabetes.

**RESULTADOS:**

Indivíduos não diabéticos apresentaram significativamente mais biópsias sem anormalidades histológicas, em relação a esteatose (42,6% *versus* 25,5%; P = 0.04), fibrose (60,6% *versus* 36,2%; P = 0.0042) e esteato-hepatite (27,3% *versus* 12,8%; P = 0.0495); os diabéticos apresentaram uma frequência significativamente mais alta de formas moderadas de esteatose (36,2% *versus* 20%; P = 0.0307) e fibrose (23,4% *versus* 4%; P = 0.0002).

**DISCUSSÃO:**

O DM-2 associou-se a formas mais avançadas de doença gordurosa do fígado dentro da população estudada. A doença gordurosa não alcoólica do fígado foi associada previamente com formas graves de doença cardíaca.

**CONCLUSÃO:**

A busca e detecção precoce da doença gordurosa em populações de alto risco são importantes para evitar o futuro desenvolvimento de formas graves e a necessidade de transplante hepático.

## INTRODUCTION

Nonalcoholic fatty liver disease (NAFLD) has been increasingly diagnosed worldwide and is now recognized as a source of public health concern. It encompasses a wide spectrum of histological features that range from simple steatosis to severe forms of fibrosis, steatohepatitis and even cirrhosis.^1^^,^^2^^,^^3^ Its pathogenesis is not completely understood, but several abnormalities have been strongly linked to its onset, such as central obesity, insulin resistance, chronic inflammation, increased uptake of fatty acids by the liver and lipotoxicity.^4^^,^^5^

NAFLD has been considered to be a hepatic manifestation of metabolic syndrome (MetS) and, as such, is strongly related to type 2 diabetes mellitus (T2DM). Some studies have correlated the severity of insulin resistance with the development of severe forms of NAFLD. Moreover, NAFLD has been described as a reliable predictor of development of T2DM.^6^^,^^7^^,^^8^^,^^9^^,^^10^

Bariatric surgery has become the standard treatment for morbid obesity, and Roux-en-Y gastric bypass (RYGB) is the surgical technique most performed worldwide nowadays.^11^ Among the morbidly obese individuals who undergo surgery, NAFLD presents high prevalence, and liver biopsy during surgery is considered mandatory in order to address the severity of NAFLD. Several studies have observed significant improvement in liver histology following RYGB, including complete reversal of liver abnormalities.^12^^,^^13^^,^^14^^,^^15^^,^^16^^,^^17^^,^^18^

## OBJECTIVE

This study sought to assess the liver histology observed in a group of individuals who underwent Roux-en-Y gastric bypass, in order to identify possible differences regarding NAFLD, between diabetic and non-diabetic individuals.

## METHODS

This was a cross-sectional study in which individuals who underwent open Roux-en-Y gastric bypass (RYGB) surgery at Hospital de Clínicas, Universidade Estadual de Campinas (Unicamp), between 2011 and 2013, were enrolled. This study was submitted to and was approved by the local Research Ethics Committee. RYGB was indicated in accordance with the American National Institutes of Health Consensus Statement criteria. Thus, surgery was indicated for individuals who had been obese for at least five years, with at least two unsuccessful attempts at conservative treatment, with a body mass index (BMI) greater than or equal to 40 kg/m^2^, or greater than or equal to 35 kg/m^2^ if this was associated with obesity-related comorbidities.^19^ The sample size was estimated using a single-proportion formula with a 95% confidence interval. The precision was set at 5% and the sample size thus calculated was 169. The inclusion criteria were that the subjects needed to be between 18 and 65 years of age, and to have undergone RYGB. The exclusion criteria comprised membership of vulnerable groups (mentally ill, institutionalized or aged below 18 years); recent or previous abuse of alcohol; antecedents of acute or chronic viral hepatitis; serological abnormalities relating to the hepatitis B or C virus; previous biliary obstruction; and preoperative biochemical examination data not completely available.

Out of the 302 subjects considered, 197 were selected for this study. The reasons for exclusion were: incomplete preoperative biochemical examinations in the medical records (77 individuals), previous alcohol abuse (19), serological abnormalities relating to chronic viral hepatitis (8) and recently treated biliary obstruction caused by gallstones (1). The main characteristics regarding demographics and anthropometric parameters were assessed.

The presence of T2DM was assessed in accordance with the criteria defined by the International Diabetes Federation (IDF) guidelines. Thus, the presence of T2DM was defined by the presence of any of the following abnormalities: fasting plasma glucose ≥ 126 mg/dl; 75 g oral glucose tolerance test with two-hour plasma glucose ≥ 200 mg/dl; glycated hemoglobin (HbA1c) ≥ 6.5%; and random plasma glucose ≥ 200 mg/dl in the presence of classical diabetes symptoms.^20^

NAFLD was assessed through histological examination of liver biopsies carried out during the surgical procedure. Liver abnormalities were classified into three categories:

1. steatosis;
2. fibrosis; and
3. steatohepatitis.

Each category was divided according to the degree of severity:

1. absent;
2. mild;
3. moderate; and
4. severe.

### Statistical analysis

The data were examined to ascertain whether the distribution was normal, in accordance with the Shapiro-Wilk test. The chi-square test and Fisher’s exact test were used to compare proportions. The Mann-Whitney test was used to compare continuous measurements between independent groups. The significance level used was 5% (P-value < 0.05). The analysis was performed using the Statistical Analysis System (SAS) software for Windows, version 9.2.

## RESULTS

Out of the 197 patients selected for this study, 155 (78.7%) were female and 42 (21.3%) were male. The mean age at the time of surgery was 38.4 years (range, 18-64). The main baseline characteristics of the subjects are summarized in Table 1.

**Table 1.**
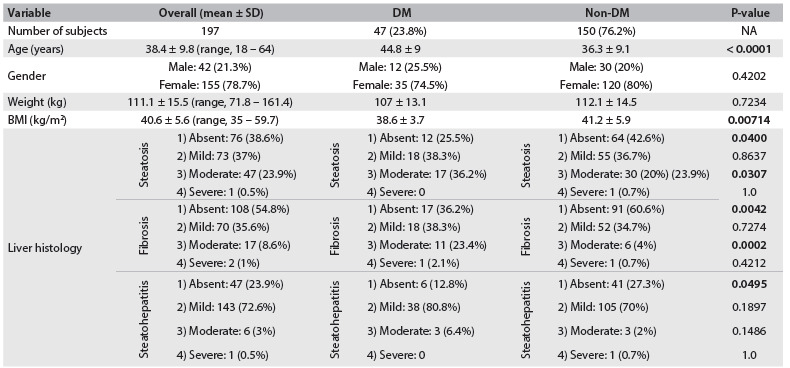
Characteristics of subjects and comparison of diabetics versus non-diabetics

DM = diabetes mellitus; SD = standard deviation; BMI = body mass index; NA: not applicable.

The diabetes group comprised 47 individuals (23.8%). Diabetics presented significantly older age (44.8 years ± 9 versus 36.3 ± 9.1 years; P < 0.0001) and lower body mass index (44.8 ± 9 kg/m^2^ versus 36.3 ± 9.1 kg/m^2^; P = 0.00714). Weight and gender distribution did not significantly differ between the groups (Table 1).

Non-diabetics presented significantly more biopsies without any abnormalities regarding steatosis (42.6% versus 25.5%; P = 0.0400), fibrosis (60.6% versus 36.2%; P = 0.0042) and steatohepatitis (27.3% versus 12.8%; P = 0.0495), while diabetics presented significantly higher frequency of moderate forms of steatosis (36.2% versus 20%; P = 0.0307) and fibrosis (23.4% versus 4%; P = 0.0002). The frequency of the mild forms did not differ in relation to any parameter in the groups. There is a detailed summary of the histological findings in Table 1.

## DISCUSSION

T2DM is currently considered to be intrinsically linked to NAFLD. The exact pathophysiological pathways through which these conditions are interconnected are not completely understood, but several metabolic abnormalities are present in both of them, especially insulin resistance, defective glucose and lipid homeostasis and chronic inflammation.^6^^,^^7^^,^^8^^,^^9^^,^^10^

Since liver biopsies during bariatric surgery are considered mandatory, since they can be performed safely and provide useful information, significantly large numbers of subjects may be analyzed within this context regarding NAFLD.^21^^,^^22^^,^^23^ On the other hand, in non-surgical settings, liver biopsies are not risk-free and therefore are not widely available. In these situations, noninvasive models that use simple laboratory examinations to assess NAFLD have been developed and validated, and may be of great usefulness for screening purposes. Of these, the NAFLD fibrosis score is the most widely known nowadays.^24^^,^^25^^,^^26^^,^^27^

This study showed that T2DM was significantly associated with the presence of NAFLD in a morbidly obese population. Furthermore, it was also associated with advanced forms of NAFLD, especially the presence of nonalcoholic steatohepatitis (NASH). The prevalence of NAFLD among obese adults with T2DM has been estimated to be greater than 70%. Alanine aminotransferase levels have been noted to be more than twice the normal levels in 20% of children with T2DM, and this is attributed to NAFLD in most cases.^28^^,^^29^^,^^30^

It is important to emphasize that the diabetic population of this study was significantly older than the non-diabetic population. Thus, these individuals may have been exposed to insulin resistance-related metabolic abnormalities for longer periods, possibly leading to liver disease of greater severity and to T2DM development from a previous MetS background.

The lower BMI of the diabetic population, compared with the non-diabetic population was also notable. This may have been due to the fact that surgery is indicated for obese individuals who are free of comorbidities when BMI is greater than or equal to 40 kg/m^2^. However, the limitations of BMI alone as an index for obesity need to be borne in mind. Since insulin resistance and its main clinical conditions (MetS and T2DM) are more related to central/visceral obesity, BMI may not be so reliable for address the severity of obesity within this group, in comparison, for example, with waist circumference or waist-to-hip ratio.^20^

The interconnection between NAFLD and T2DM has been noted previously in the literature. Fukuda et al. observed that even among non-overweight individuals, NAFLD was an independent predictor of T2DM.^31^ Yamazaki et al. followed up a cohort of 4,604 subjects and showed that, in a long-term evaluation of individuals with ultrasonographically assessed liver disease, that improvement of NAFLD leads to reduced incidence of T2DM.^32^

NAFLD in diabetic individuals has been linked to severe complications of heart disease. Targher et al. demonstrated that NAFLD was independently associated with cardiovascular events, and therefore suggested that NAFLD was not merely a marker of cardiovascular disease (CVD) but might also be involved in its pathogenesis. The possible molecular mediators linking NAFLD and CVD include proatherogenic mediators released from the liver, including C-reactive protein, fibrinogen and plasminogen activator inhibitor-1.^33^ Mantovani et al. observed that NAFLD is independently associated with early left ventricular diastolic dysfunction in type 2 diabetic patients with preserved systolic function.^34^

Since bariatric surgery provides significant weight loss and high rates of resolution of metabolic comorbidities, it has also been associated with improvement of liver disease. It is now believed that this metabolic improvement is only partially linked to the weight loss itself. In fact, it begins very early following surgery, possibly due to structural changes in gastrointestinal transit, leading to increased incretin/adipokine activity and immunomodulation. Even individuals with severe forms may benefit from surgical outcomes. Considering the possible ominous risks of NAFLD/NASH, especially end-stage liver disease and liver cancer, this benefit is even more pronounced.^12^^,^^13^^,^^14^^,^^15^^,^^16^^,^^17^^,^^18^^,^^35^^,^^36^^,^^37^

This study has some limitations, especially its use of data collected from the medical records, which may provide data of poor quality and may lead to the impossibility of evaluating a considerable number of individuals, because of incomplete medical reports. In this study, incomplete medical records caused high loss of subjects who might have been suitable for analysis. Furthermore, there were only a few subjects with severe forms of NAFLD within the sample studied.

Since NAFLD is nowadays the third most common indication for liver transplantation in the USA, and it is expected to be the first by 2030,^38^^,^^39^ screening and early detection of high-risk populations, such as diabetics, may have a major impact on its onset and further prognosis.

## CONCLUSION

Liver disease occurred more frequently among diabetics in this study. T2DM was associated with more advanced forms of NAFLD.
